# Effectiveness of a nurse-led case management home care model in Primary Health Care. A quasi-experimental, controlled, multi-centre study

**DOI:** 10.1186/1472-6963-8-193

**Published:** 2008-09-23

**Authors:** JM Morales-Asencio, E Gonzalo-Jiménez, FJ Martin-Santos, JC Morilla-Herrera, M Celdráan-Mañas, A Millán Carrasco, JJ García-Arrabal, I Toral-López

**Affiliations:** 1 Andalusian School of Public Health, Granada, Spain; 2 Healthcare District, Andalusian Healthcare Service, Málaga, Spain; 3 Andalusian School of Public Health, Granada, Spain; 4 Healthcare District, Andalusian Healthcare Service, Almería, Spain; 5 Healthcare District, Andalusian Healthcare Service, Granada, Spain

## Abstract

**Background:**

Demand for home care services has increased considerably, along with the growing complexity of cases and variability among resources and providers. Designing services that guarantee co-ordination and integration for providers and levels of care is of paramount importance. The aim of this study is to determine the effectiveness of a new case-management based, home care delivery model which has been implemented in Andalusia (Spain).

**Methods:**

Quasi-experimental, controlled, non-randomised, multi-centre study on the population receiving home care services comparing the outcomes of the new model, which included nurse-led case management, versus the conventional one. Primary endpoints: functional status, satisfaction and use of healthcare resources. Secondary endpoints: recruitment and caregiver burden, mortality, institutionalisation, quality of life and family function. Analyses were performed at base-line, and at two, six and twelve months. A bivariate analysis was conducted with the Student's t-test, Mann-Whitney's U, and the chi squared test. Kaplan-Meier and log-rank tests were performed to compare survival and institutionalisation. A multivariate analysis was performed to pinpoint factors that impact on improvement of functional ability.

**Results:**

Base-line differences in functional capacity – significantly lower in the intervention group (RR: 1.52 95%CI: 1.05–2.21; p = 0.0016) – disappeared at six months (RR: 1.31 95%CI: 0.87–1.98; p = 0.178). At six months, caregiver burden showed a slight reduction in the intervention group, whereas it increased notably in the control group (base-line Zarit Test: 57.06 95%CI: 54.77–59.34 vs. 60.50 95%CI: 53.63–67.37; p = 0.264), (Zarit Test at six months: 53.79 95%CI: 49.67–57.92 vs. 66.26 95%CI: 60.66–71.86 p = 0.002). Patients in the intervention group received more physiotherapy (7.92 CI95%: 5.22–10.62 vs. 3.24 95%CI: 1.37–5.310; p = 0.0001) and, on average, required fewer home care visits (9.40 95%CI: 7.89–10.92 vs.11.30 95%CI: 9.10–14.54). No differences were found in terms of frequency of visits to A&E or hospital re-admissions. Furthermore, patients in the control group perceived higher levels of satisfaction (16.88; 95%CI: 16.32–17.43; range: 0–21, vs. 14.65 95%CI: 13.61–15.68; p = 0,001).

**Conclusion:**

A home care service model that includes nurse-led case management streamlines access to healthcare services and resources, while impacting positively on patients' functional ability and caregiver burden, with increased levels of satisfaction.

**Trial registration:**

ISRCTN44054549

## Background

The complexity and demand for home care services has grown over the last 20 years. In Spain, 57% of homes have at least one person requiring care, and in 66% of cases the family is the sole caregiver [1]. Home care services provided to elderly people and chronically ill patients is one of the most important challenges facing our Healthcare Systems.

Reportedly, home care demand is related to co-morbidity, functionality for daily living, perceived health status and previous demand for health services [2]. But the term "home care" refers to a broad range of services and depending on the various contexts and countries, there is great variability among providers, target populations, services and duration of home care services [3].

Systematic reviews addressing home care for the elderly and chronically ill have reported mixed conclusions. Multi-dimensional assessment and preventive visits, have led to a decrease in institutionalisation and improvements in patients' functional status [4,5], with additional beneficial effects when assistive technologies are used [6].

Risk minimisation at the homes of elderly people by different providers has also proved effective [7]. Indeed, interventions targeting caregivers have led to improvements in their knowledge, psychological wellbeing or depression status [8-10].

But one of the main problems in devising and delivering services to these population groups stems from the broad range of resources and providers involved. There are major differences among professionals and settings, and co-ordination of care is not always optimal [11]. Varying outcomes have been reported for planning and co-ordination for discharges on the part of hospitals, using approaches such as disease or case management [12-15]. Results depend on the different sub-groups of patients considered, but mainly such approaches had a positive impact on re-admissions and, in some cases, even led to lower mortality. Because the home care services available are so scattered, many studies have been conducted in order to co-ordinate and provide a comprehensive service. Case management has been one of the most widespread initiatives adopted in different countries towards this end. The heterogeneity of interventions and organisations have, to date, referred to a broad range of outcomes and patients [16,17].

In general terms, case management is defined as a collaborative process of assessment, planning, facilitation and advocacy for options and services to meet an individual's health needs through communication and available resources, thus promoting quality, cost-effective outcomes [18].

There are five major areas of intervention in case management: screening of the population at risk; providing timely access to information about social and health resources and how to access them; providing support for informed decision-making; facilitating the integration of multiple services; and ensuring efficient allocation of the scarce resources available, as well as maximizing continuity of care [19].

In Spain, attempts have been made to implement initiatives intended to achieve excellence in home care services [20,21]. The most recent was the creation of a whole new system within Primary Health Care that includes advanced practice nurses in Southern Spain.

From this standpoint, a two-tier decision-making model was incorporated into traditional home care services. The first level entails direct decisions by general practitioners and family nurses, while a second level is led by case management nurses. Since 2002, over 300 case management nurses were deployed throughout the entire region of Andalusia to provide care for seven million inhabitants. In two years alone, case managers had performed 46,676 assessments [22].

This new model of care required evaluation given that the vast majority of studies had been performed in other countries where Health Care Services differ greatly from the system in Spain. This research was designed primarily to describe the outcomes for home care patients and their caregivers, in terms of functional status, use of social and health resources and satisfaction, comparing the new and conventional models. As for our secondary aims, we also tried to determine the effects on caregivers, institutionalisation, mortality, quality of life, family function and to describe the profile of caregivers in our region.

## Methods

### Design

Quasi-experimental, prospective, multi-centre study, with a concurrent control group, conducted between 2003 and late 2006, focusing on public home care services delivered by Primary Health Care in four districts in Andalusia (Spain), namely Malaga (DSM), Almeria (DSA), Granada (DSG) and Costa del Sol (DSCS).

### Study population

patients and caregivers initiating the Home Care (HC) programme from the Andalusian Healthcare Service and targeting some of the following population sub-groups: 1) terminally ill patients with advanced stage, progressive, incurable, multi-symptomatic disease with no reasonable chance of responding to specific treatment, with estimated survival not exceeding six months; 2) dependent patients who require assistance for their daily activities (Activities of Daily Living, ADL) and are immobilised at home, namely subjects not included in sub-group (1) who, for whatever cause, are forced to spend most of their time in bed and/or require help to move, which prevents them from leaving home, except for rare exceptions; 3) patients not included in sub-groups (1) and (2), recently discharged from hospital, requiring home care during a short period of time, most likely for under two months; (4) main caregivers for any of the patients described in the previous sub-groups. These criteria are established by the Andalusian Healthcare Service and they were not modified in order to obtain patients in a "real-practice" situation.

### Inclusion criteria

Patients and main caregivers were allocated to either the intervention or control group, depending on whether or not they had access to home care services delivered in line with the new model by their Healthcare Centre. As the new model was initiated in 2002 with a progressive implementation along the Districts, a time-window of several years with Health Centres in the traditional model and others with the new one was set. During 2002–2006, both models co-existed and let us to evaluate with a non-randomized approach the effectiveness of the new service. The period for enrolment of subjects and inclusion in the sample started at the various healthcare centres six months after case management nurses initiated the programme. This gave nursing staff sufficient time to adapt to their new functions and roles.

### Exclusion criteria

a. Institutionalisation or change of residence to an area not covered by the study, thus preventing the minimum follow-up required.

b. Hospitalisation for longer than seven days, (except for terminally ill patients who were readmitted for disease stabilisation and symptom control, as part of their usual process of care). This criterion was established in order to avoid the influence of hospitalization interventions and outcomes.

With these criteria in mind, the population potentially requiring home care services in the healthcare districts under the scope of the study was estimated at 1,032,333. Malaga with 50.93% of the total potential population was in a position to contribute more subjects to the study, followed by the Costa del Sol district with 20.46%, Almeria with 20.6% and lastly Granada with 8.55%.

### Sampling

We conducted stratified, consecutive sampling of all patients initiating the programme, first by district, secondly by healthcare centre and thirdly by group of home care services.

Assuming an alpha risk of 0.05 and beta risk of 0.20 in bilateral contrast, the calculated sample size required 143 subjects in the first group and an equal number in the second in order to detect differences equal to or higher than 15 units in functional capacity measures (Barthel scale, range 0–100). We assumed a common standard deviation of 35 for this parameter, as identified by Landi et al. in their study [23]. We estimated a follow-up drop-out rate of 40% given the special features of the elderly and dependent population, with high probabilities of institutionalisation or change of address. Sample calculations were performed using the Granmo 5.2 application.

### Follow-up periods

The following follow-up periods were established depending on the characteristics of the population. For the terminally ill, this was up until time of death; for patients with mobility problems, six and twelve months; for post-hospitalisation patients, up to two months (the time estimated for recovering their functional status; if not, they were included in the immobilised patients in the course of normal attention); for family caregivers, the time frame established for the sub-groups of the subject receiving care, plus an additional six-month period in the event of death (in order to provide grief support).

The list of variables and measures are described in Table 1.

**Table 1:** Variables and measures

**CHARACTERISATION VARIABLES**	
1. Healthcare centre	
2. Age	
3. Patient and caregiver gender	
4. Family relationship between patient and caregiver	*1. Spouse/2. Son-Daughter/3. Brother-sister/4. Others*
5. Home care delivery sub-programme which the subject belongs to	*1. Immobilised patients/2. Patients recently discharged form hospital requiring home care/3. Terminally ill patients/4. Caregiver for any of these patients*
6. Number of daily hours devoted to care by caregiver	*< 1 hour/1–2 hours/3–5 hours/6–8 hours/10–20 hours/>20 hours (Thresholds from the Aged People Characteristics Study)*
7. Patient and caregiver's health problems	*Classification of health problems from the National Health Survey*
8. Programme start and end dates	

**VARIABLES RELATED TO CLINICAL OUTCOMES AND QUALITY OF LIFE (DEPENDENTS):**	

1. Patient's functional capacity	Barthel and Lawton-Brody scales
2. Caregiver burden	Zarit test
3. Family role/function	APGAR family test
4. Cognitive function	Pfeiffer test
5. Quality of life	EUROQOL 5D
6. Satisfaction	SATISFAD, specific questionnaire validated for assessment of satisfaction with home care services [38]
7. Institutionalisation	
8. Mortality	
9. Management of therapeutic regimen	Nursing Outcomes Classification code 1813 [39]

**VARIABLES RELATED TO USE AND DELIVERY OF SERVICES (DEPENDENTS)**	

1. Hospital admissions	
2. A&E visits	
3. home visits by nurse	
4. social worker interventions	
5. physiotherapy sessions	
6. caregiver visits to healthcare centre	
7. patient visits to healthcare centre	

### Characteristics of home care services delivered to the intervention and control groups

#### Population

It was the same for both groups (Subjects requiring care after hospital discharge, immobilised subjects with serious difficulties to leave home, patients with advanced terminal illness and expected survival under six months and non-professional caregivers assisting subjects with delivery of home care).

#### Professionals

In the control group, the main providers were the community nurses and general practitioners (GPs), with the support of social health workers, physiotherapists and occupational therapist. This was the usual staff of the Health Centre and eventually, in the same terms of any citizen belonging to the Centre, the patients could be referred to specialist consultation by their GPs along the course of their health process. In the intervention group, the team was the same except the addition of the nurse case manager. This nurse had advanced roles and a specific selection process, with higher qualification and salaries, according to their responsibilities.

The clinical recording system was the same for both groups (computerized in Health Centre and paper recording in homes). Case managers had in addition a mobile phone for a better accessibility for patients and caregivers.

Activities and services: the patients in the home care services were included in each subgroup by clearly defined criteria described in clinical protocols existing in the Andalusian Health Service. These criteria are used in daily work in all the Health Centres and are well-known by GPs and nurses. These criteria were maintained in the Centres with the new model (case management).

The patients had follow-up visits accordingly with their health demands, mainly delivered by the community nurse who coordinates with other members of the Health Centre team if required. The focus of the service was giving support to patients and caregivers. This care was common to the control and intervention group.

In addition to this, in the intervention group the case manager reviewed the target population census, delivered home care visit with comprehensive assessment and detection of needs upon request from team members, established co-ordination mechanisms with other institutions and professionals (included the Hospital level), arranged technical assistance at home, carried out specific activities with caregivers (i.e.: group workshops for emotional and care giving support), took part in commissions for ongoing assistance and provided tele-care through telephone proactive follow-up.

### Data compilation

Measures were adopted to avoid interfering with nurses' normal practice conditions, both in the intervention and control groups. We established a progressive register of patients enrolled in the study. For the intervention group, two sources of data were used, namely (1) the patient (and/or caregiver) – data was recorded directly using a sheet designed *ad hoc *which was completed by the case management nurse in the course of home visits – and (2) the information system in place at each healthcare centre – which was examined by two interviewers with a nursing background, who were specifically trained for data compiling. For the control group, patient and/or caregiver data were gathered by telephone surveys and self-completion questionnaires that were returned by post by the interviewers themselves. The aim here was to avoid any interference in nurses' daily practice and the Hawthorne effect, since staff were aware that they were being compared to case management nurses at the healthcare centres where the new model had been implemented. Interviewers were trained to structure telephone interviews using the Filemaker Pro 6.0 data-base. This was designed as a tool both to structure the interviews and for data compilation. A pilot study with twenty patients-not included in the study-was run before the study *per se *began. For both groups, a third party, who had not taken part in the data compilation process, keyed the variables into the statistical data-base.

We checked that all the main outcome variables had been validated for telephone interviews by various different studies in order to guarantee validity of data compilation in the control group [24]: Barthel scale [25] (intra-class correlation coefficient with the in situ version 0.89), Pfeiffer test [26] (sensitivity and specificity of 0.74 and 0.79 respectively), Lawton-Brody index [27]. The APGAR family test had not been validated for telephone use, but we decided to retain this test since it addressed a secondary goal. The Zarit and EUROQOL 5D test versions had been validated for self-completion.

All patients and caregivers were asked to give their written consent to take part in the study; for patients with cognitive decline, consent was requested from the individual's main caregiver. The study was approved by the Ethics Committees of both the Andalusian School of Public Health and the various healthcare districts taking part. The software package SPSS 13.0 was used for statistical analysis.

### Data Analysis

The analysis was structured as follows:

- Descriptive statistics for characterisation and outcome variables, via exploratory analysis, using central trend and scatter measures for quantitative variables and analysis of proportions for qualitative variables.

- Analysis of the type of distribution and normality test for each variable using the Kolmogorov-Smirnov and Shapiro-Wilk tests, together with Q-Q normal probability plots.

- Bivariate analysis using Student's t-test for means in normal distribution variables (using the Levene test for variance equality) and non-parametric tests such as the U Mann-Whitney test (independent samples) and Wilcoxon test (paired data) for variables showing non-normal distribution. For qualitative variables we used the chi squared test, and Fisher's exact test whenever necessary for each contingency table. We also used correlation and regression measures when necessary for quantitative variables.

For bivariate analysis of qualitative and quantitative variables we used ANOVA with the post-hoc Scheffe contrast method and analysis of fixed and random effects.

For comparison of final outcomes – survival, institutionalisation – we devised survival models using the Kaplan-Meier procedure and log-rank analysis for comparison of groups. Furthermore, whenever necessary, results were described in terms of risk (relative risk (RR)/odds ratio (OR)) or by their corresponding 95% confidence intervals.

- Multivariate analysis: we used multiple linear regression and logistic regression depending on whether the dependent variable was continuous or dichotomous.

- In all cases, the lowest acceptable alpha error was 0.05.

- We conducted intention to treat analyses.

## Results

**Figure 1:**
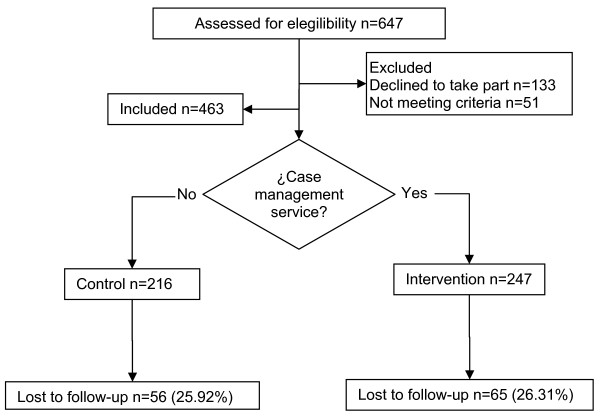
The sequence for selection, recruitment and follow-up of the sample.

The number of subjects included in the survey totalled 463 out of the minimum number of 286 required according to initial estimates. The sequence for selection, recruitment and follow-up of the sample is explained in Fig. 1. Throughout the process, 26.1% of subjects were lost to follow-up, leaving a total of 342 subjects for analysis. The most frequent cause for withdrawal from the study was death – 17.70% of the total – and there were no differences in terms of losses in the two groups included in the study (p = 0.659).

Thirty-eight healthcare centres in the four districts took part in the survey, from a total of 45 centres that could have contributed to the sample. The distribution of the sample through Districts was: Málaga (277; 59.80%) Granada (101; 21.8%), Almería (72; 15.6%) y Costa del Sol (13; 2.8%). The sample was mostly made up of caregivers and mobility-dependent patients (44.5% and 35.4%, respectively), followed by post-hospitalised subjects (15.1%) and terminally ill patients (5%). Patient and caregiver characteristics are detailed in Tables 2 and 3; there were no significant differences between groups.

**Table 2:** Patient characteristics

	**Intervention (n = 130)**			**Control (n = 128)**			**Sig.**
	
		**Mean (SD) or n/N**	**95%CI or %**		**Mean (SD) or n/N**	**95%CI or %**	**p**
Age		75.36 (13.16)	(73.20–77.79)		77.24(13.06)	(74.94–79.53)	NS
Men		53/129	41.10%		41/125	32.80%	NS

**Length of home care (subgroups)**							

	n	Intervention		n	Control		

Immobilised (n = 165)	75	522.68(281.22)	450.03–595.33	90	696.64(271.64)	639.09–754.20	NS
Terminal care (n = 23)	13	137.69(76.05)	91.73–183.65	10	162.44(34.32)	136.05–188.82	
Hospital discharge (n = 70)	42	65.07(13.05)	61.00–69.14	28	62.57(7.01)	59.85–65.29	

**Main health problems (patients)**							

	Intervention (299 cases*)			Control (255 cases*)			
Problems*	n (%)			n (%)			p

Cardiovascular	81(27.09)			81 (31.76)			0.052
Endocrine	53(17.2)			39 (15.29)			
Neurologic	40(13.7)			42(16.47)			
Injury	38(12.0)			25(9.80)			
Bone and joint	32(10.70)			39(15.29)			
Prosthetic & renal	29(9.69)			10(3.92)			
Respiratory	26(8.69)			19(7.45)			

*The same patient could have several concomitant problems

**Table 3:** Main caregivers' characteristics

	**Intervention (n = 117)**		**Control (n = 88)**		Sig.
	**Mean (SD) or n/N**	**95%CI or %**	**Mean (SD) or n/N**	**95%CI or %**	**p**

Age	57.24(13.31)	(54.67–59.80)	58.35(9.78)	(55.60–61.10)	NS
Women	101	86.32%	60	68.18%	NS

**Daily time for care**					

Missing data	6	5.10%	29	33.30%	
< 3 hours/day	2	1.70%	3	3.30%	
3–5 hours/day	7	6.00%	8	9.10%	
6–8 hours/day	20	17.10%	8	9.10%	
9–20 hours/day	38	32.50%	20	22.70%	NS
> 20 hours/day	44	37.60%	20	22.70%	

**Patient-caregiver relationship**					

Spouse	41	35.00%	14	15.90%	NS
Son/Daughter	61	52.10%	40	45.45%	
Other	15	12.90%	7	7.95%	
Missing data	0	0	27	30,70%	

Subjects receiving home care services were generally females aged around 76 years, with cardiovascular and metabolic health problems, a high degree of mobility-related dependency and who required considerable assistance.

The profile for main caregivers is a female – patient's daughter or spouse – around 57 years of age, who devotes over twenty hours a day to care. These carers also suffer from chronic bone & joint and cardiovascular problems. At the initial stages, the care-related tasks triggered anxiety and a high risk of exhaustion in carers.

Both, patients and caregivers' profiles, were representative from the usual patients attended in the Home Care Programme in Andalucía (confirmed with the profiles available at the Andalusian Healthcare Service Annual Reports).

Results obtained from multi-dimensional assessment of patients overall highlighted a high level of functional decline, both in terms of performing essential activities of daily living and instrumental tasks: average Barthel index 48.84 (SD 32.44), average Lawton-Brody 1.92 (SD 2.09) and average Pfeiffer test 2,64 (SD 3.13). Furthermore, initial caregiver burden was high (average value in the Zarit test 58.50; SD 14.8), at the lower limit for the range of intense burden. Despite stamped, addressed envelopes and telephone reminders, the response rate for quality of life was not high enough for any analysis to be conducted.

According to the Barthel index, functional capacity showed significant base-line differences, i.e. ten points lower in the intervention group vs. the control group (RR: 1.52 95%CI: 1.05–2.21; p = 0.0016). These differences disappeared at six months (RR: 1.31 95%CI: 0.87–1.98; p = 0.178). Due to the heterogeneity of groups, we developed analysis of sub-groups (Table 4). In the main cluster of patients (immobilized), the mean differences in functional status measured with Barthel Index at baseline were important: 39.19 (95% CI: 32.54–45.83) in the intervention group, versus 50.00 (95% CI: 42.79–57.21; p = 0.021) in the control group. At six months, these clinical and statistically significative differences, were reduced slightly (43.15; 95% CI: 34.66 to 51.63; vs. 50.62; 95% CI: 43.06–58.18; p = 0.222). The Lawton-Brody and cognitive status remained with important differences.

**Table 4:** Multidimensional assessment of patients' subgroups (at base-line, 2 & 6 months)

	**Immobilised (n = 165)**					
	
	Baseline			6 months		
	
	Mean (95%CI)		p	Mean (95%CI)		p
	
	Intervention(n = 75)	Control (n = 90)		Intervention(n = 75)	Control (n = 90)	
Family APGAR	8.02 (7.41–8.62)	8.88 (8.35–9.40)	0.023*	8.50 (7.94–9.06)	9.05 (8.63–9.47)	0.142
Barthel	39.19 (32.54–45.83)	50.00 (42.79–57.21)	0.021*	43.15(34.66–51.63)	50.62 (43.06–58.18)	0.222
Pfeiffer	3.89 (2.97–4.80)	2.34 (1.64–3.04)	0.042*	4.13(3.10–5.15)	2.13 (1.47–2.79)	0.008*
Lawton-Brody	1.13 (0.78–1.47)	2.11 (1.64–2.58)	0.008*	1.08(0.71–1.46)	2.14 (1.62–2.65)	0.007*

	**Hospital discharges (n = 70)**					
	
	Basal			2 months		
	
	Mean (95%CI)		p	Mean (95%CI)		p
	
	Intervention(n = 42)	Control (n = 28)		Intervention(n = 42)	Control (n = 28)	

Family APGAR	8.90 (8.30–9.47)	9.45 (8.90–9.96)	0.217	9.29 (8.79–9.79)	9.53 (8.95–10.00)	0.378
Barthel	47.38 (38.51–56.25)	66.79 (52.06–81.51)	0.004*	70.44 (60.39–80.50)	71.35 (56.80–85.89)	0.678
Pfeiffer	2.10 (1.05–3.15)	1.00 (0.00–2.07)	0.090	1.65 (0.48–2.81)	1.00 (0.08–1.81)	0.407
Lawton-Brody	1.68 (1.12–2.24)	3.39 (2.23–4.56)	0.006*	3.03 (1.94–4.13)	3.92 (2.46–5.39)	0.335

	**Terminal care (n = 23)**					
	
	Basal			6 months		
	
	Mean (95%CI)		p	Mean (95%CI)		p
	
	Intervention(n = 13)	Control (n = 10)		Intervention(n = 13)	Control (n = 10)	

Family APGAR	8.00 (6.31–9.69)	9.00 (6.85–11.15)	0.371	NA	NA	-
Barthel	59.62 (36.89–82.34)	47.22 (13.71–80.73)	0.647	NA	NA	-
Pfeiffer	3.27 (0.55–5.99)	0.20 (0.00–0.76)	0.090	NA	NA	-
Lawton-Brody	2.08 (0.81–3.35)	2.56 (0.38–4.73)	0.794	NA	NA	-

In the post-hospitalised patients the results showed that they achieved greater recovery rates – the base-line average Barthel score was significantly lower in the intervention group (47.38; 95%CI: 39.68–55.08; vs. 66.79; 95%CI: 54.05–79.52; p = 0.004), with considerable improvement at two months, by which time any differences disappeared (70.44; 95% CI: 61.73–79.15; vs. 71.35; CI 95%: 58.79–83.91; p = 0.678).

Given high mortality rates and, hence, a loss of statistical power, we were unable to assess functional ability at twelve months. Table 4 shows the results of base-line multi-dimensional assessment at two and six months.

In order to ascertain which factors have the greatest impact on recovery of post-hospitalised patients' functional capacity, we devised a multiple linear regression model, where the dependent variable was the Barthel score at two months, along with number of rehabilitation, occupational therapy and social worker interventions. However, we failed to identify a valid model to explain the result (R^2 ^= 0.37; p = 0.057).

We later devised a second model, based on the hypothesis focusing on a subject's potential capacity, and on how, by enhancing resources such as strength (with the support of technical aid), knowledge and willpower, patients can achieve greater independence to meet their own needs following hospital discharge. We included the following predictive variables: NOC outcome criterion "1813 Therapeutic Regimen Management", at two months (this criterion includes the patient's description of her/his own responsibilities in care and treatment, of the desired effects of care, the course of the disease, or conducting self-monitoring techniques); age of main caregiver (as the determining factor for support); patient age; total number of visits; number of technical aids obtained; and number of interventions by social workers. Table 5 describes the results of this regression. It also shows how understanding of the therapeutic regimen and access to technical aid are the factors with the greatest impact on recovery of functional capacity at two months post hospital discharge. The model met the assumption regarding independence of residuals (Durbin-Watson statistical value of 1.65) and accounts for approximately 89% of the functional capacity figures obtained at two months.

**Table 5:** Linear regression model: factors influencing the recovery of functional status after hospital discharge

**MODEL**					
R^2^	Standard Error	Sig.	Durbin-Watson		
0.897	10.217	0.0001	1.953		

**ANOVA**	Sum Squares	Df	F	Sig.	

Regression	16327,92	6	26,07	0.0001	
Residual	1878,85	18			
Total	18206,78	24			

				95%CI	
**FACTORS**	Beta (Standardised)	t	Sig.	Lower	Upper

(Constant)		2.103	0.050	0.039	77.786
NOC1813 (Understanding of Therap. Regimen) (n = 60)	0.609	6.364	0.0001	11.362	22.561
Patient age (n = 48)	-0.455	-4.512	0.0001	-1.350	-0.492
Caregiver age (n = 48)	0.572	6.232	0.0001	0.723	1.458
No. Visits (nurse and case manager) (n = 55)	-0.532	-6.401	0.0001	-3.230	-1.634
No. Social aids (n = 45)	-0.885	-7.054	0.0001	-48.122	-26.035
No. Social worker interventions (n = 33)	0.433	3.164	0.005	5.448	26.987

As to use of healthcare services, the study points to a larger number of interventions by providers such as social workers and physiotherapists in the intervention group. This group received significantly fewer visits by home care nurses – four visits less on average – than patients in the control group. Caregivers in the intervention group attended the healthcare centre considerably fewer times than those in the control group. There was a lower total number of visits for the intervention group, even when visits by the case management nurses are also included (Table 6), except for the sub-group of post-hospitalisation patients who received more visits than those in the control group (6.53; 95%CI: 4.41–8.64 vs. 4.69 95%CI: 1.18–8.20; p = 0.009).

**Table 6:** Utilisation of health resources

	Mean		
	
	Interv (n = 247)	Control (n = 216)	p
Home Visits (Community Nurse)	7.58 (6.05–9.10)	11.82 (9.10–14.54)	0.022*
Home visits (Community + CMN^1^)	9.40 (7.89–10.92)	11.80 (9.10–14.54)	0.758
Social Worker interventions	1.00 (0.75–1.25)	0.38(0.21–0.55)	0.0001*
Physiotherapist interventions	7.92 (5.22–10.62)	3.24 (1.37–5.10)	0.0001*
Hospital Re-admissions	0.75 (0.47–1.03)	0.66 (0.40–0.91)	0.599
A&E visits	2.53 (1.72–3.35)	2.24 (1.63–2.84)	0.526
Caregivers visits to the Health Centre	7.79 (5.68–9.90)	26.30 (19.19–33.41)	0.0001*

^1^CMN case management nurse

**Figure 2:**
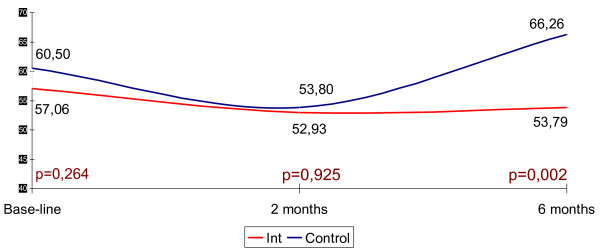
At 6-month follow-up.

Caregiver burden, as assessed via the Zarit test, showed a significant initial burden, with no differences between the control and intervention groups. Throughout follow-up, there was an improvement in Zarit scores in the post-hospitalisation patient group, although the differences remained unaltered. At 6-month follow-up, there was a moderate drop in caregiver burden in the intervention group, essentially on account of mobility-impaired patients, while there was a significant increase in the control group (Fig. 2). The high rate of non-responders at twelve months prevented analysis.

Analysis of patient survival in the control and intervention group show no statistically significant differences (RR = 0.871; 95%CI: 0.509–1.489; p = 0.682). There were no appreciable differences either between groups in terms of institutionalisation (RR = 0.857; 95%CI: 0.280–2.624; p = 0.506).

There was a significantly higher degree of satisfaction in the intervention group – 16.88 (95%CI: 16.32–17.43) vs. 14.65 (95%CI: 13.61–15.68) (p = 0.001).

## Discussion

By incorporating a model based on case management for the delivery of home care services by specifically trained nurses from the Basic Primary Care Teams (BPCTs) in Andalusia (Spain), we have been able to partially verify the hypothesis that health outcomes for patients and caregivers can be enhanced. In addition, this research has highlighted a change in the pattern of utilisation of social and healthcare services at the centres studied; current trends are characterised by greater diversification – more physiotherapy and social worker interventions, as well as telephone consultations with the case manager community nurse – together with a drop in the number of home visits and consultations at the healthcare centre.

Regarding patients' characteristics, the study highlights a greater degree of dependence and overall decline of subjects in the intervention group vs. control group at the time of enrolment in the programme. It would appear that patients suffering severe functional decline who, under the traditional model, would either be kept in hospital or would be referred from one service to another in the healthcare system, may now be referred to a professional responsible for case management at the primary level of healthcare. Also, our findings appear to indicate that the services provided by case management nurses and by community nurses are genuinely complementary. So, it appears that case management needs to be activated only in the case of patients with complex conditions requiring a broad range of services and co-ordination among healthcare professionals. The system should ensure that those services – some provided by other sectors – are readily available so that any changes in management of healthcare demand do not ultimately fall back on the flexibility of informal caregivers.

The results highlight a better recruitment rate of caregivers through the new home care model. This may suggest a trend that alters the traditional relationship between the formal and informal systems of care delivery. Here, informal care ceases to act only as a "resource" available to the formal system, and becomes acknowledged as a "client", or "co-client" of the formal healthcare delivery system [28]. The caregiver profile identified in the study, i.e. vast majority of women, patients' daughters or spouses – demonstrates the clear gender bias in caregiver role assignment; culturally, it still seems inevitable that the women most closely related to the patient should take on this role [29]. The fact that Healthcare Services are focusing their attention on this group as clients, enhances opportunities for collaboration, increases awareness of their status of inequality and their need to access resources that may lead to eradicating this inequity.

With regard to health outcomes, the study highlights first and foremost that recovery of patients' ability to perform essential activities of daily living (Basic Activities of Daily Living) is significantly greater among patients receiving care through the new model, especially among the sub-group of patients who were discharged from hospital. Given base-line differences, even bearing in mind their greater chances of improvement, the highest recovery rate indicated by the Barthel index seems to be directly related to interventions; the study shows that case management nurses are more frequently involved when patients receive more home care services such as rehabilitation and physiotherapy, and more support resources, such as technical aid. These findings are consistent with the results reported by Britton for patients who were discharged from hospital after suffering a stroke, and for which rehabilitation at home is reportedly more effective if combined with early discharge and when home care is offered to patients at a time when functional decline is greater and their transport needs more complex [30].

It is hardly surprising that no differences were found between both groups in terms of the impact of the intervention on patient survival given the profile of patients included in the study and considering that the effect of home care services on mortality tends to be appreciable only in young subjects. Hence, this should not be a goal *per se *of home care programmes, especially when targeting elderly, immobilised patients [4].

As to results in the group of caregivers, the new model appears to avoid, and even to slightly reverse the natural trend towards increased caregiver burden over time, as shown by the evolution of Zarit test scores for the control group. It is quite possible that structured support, with specific interventions which are key in the new model will be efficient in curbing or delaying further aggravation of the situation [8,31].

The results of this study highlight the new model's interesting effect on the pattern of service use. Case management seems to rationalise the use of services by bringing providers into the home care setting, such as physiotherapists or social workers, who have a lesser role in the traditional model. At the same time, the new model curbs the use of general services, such as nurse home visits – on average three visits less in the intervention vs. control group – or consultation with physicians or nurses at healthcare centres. It is very plausible that this downward trend is also linked to the possibility for patients and caregivers to access telephone assistance from case management nurses.

Among the effects of this new pattern, the lower number of caregiver visits to the healthcare centre is particularly noteworthy. For caregivers this means that they are relieved of one of their most frequent and time consuming tasks [32] (i.e. making the necessary arrangements at health services facilities to cover patient's needs); they also gain greater accessibility through telephone-based assistance. This drop in the number of visits to health centres provides clear proof that the demand can largely be managed using care options other than physician/nursing consultations, providing greater degrees of accessibility and satisfaction for the population.

One of the most important questions arising in the face of this new utilisation activity is its possible impact on satisfaction, since part of the demand is provider-induced, in this case by the case management nurse. When comparing the data, satisfaction was seen to be significantly higher in the group of patients receiving care under the new model vs. the control group, in line with other studies [33]. How can we account for better outcomes and greater satisfaction achieved with fewer visits? The key seems to lie in the diversification of providers and resources, and the availability of direct telephone assistance – proactive or reactive – which to some extent has shifted *in situ *assistance. The literature on case management reports on a myriad of experiences with different forms of tele-care that have achieved satisfactory results in randomised experimental studies [34]. Confirmation of these effects should be the focus of future research since there is currently no automatic information system available to shed light on the incidence, type of calls and interventions conducted through this approach.

This study has shown no effect on frequency of A&E visits or hospital admissions, much like other case management studies conducted in different settings [35-37]. However, there is no doubt that a considerable effect on readmissions, and even on mortality, is achieved when patient-specific interventions are implemented, as seen in the case of individuals with heart disease, for instance [12,13,15]. Still pending for the full implementation of this model is the devising of critical, specific pathways for particular patients.

Equally, no effect on institutionalisation was identified in this study, despite the fact that the benefits of performing multi-dimensional assessment of elderly patients in terms of reducing admissions to residential homes is well documented in the literature [4]. It is quite possible that cultural tradition in Andalusia has favoured care in the family setting rather than institutionalisation because, amongst other factors, the public offering of residential homes is scarce and precarious, in comparison with other neighbouring countries.

### Limitations

The size of the sample obtained after removing drop-outs has prevented us from drawing conclusions on some specific sub-groups of patients receiving the service, namely terminally ill patients and some outcomes as quality of life or follow-up to 18 months.

A complex data compilation process, not always exempt from possible bias, was required in view of the need to resort to different sources of information using *ad hoc *procedures, and so as to avoid upsetting the normal conditions of professional nursing practice, without exerting any influence on nursing staff. The different procedures used to obtain data from patients and caregivers in the intervention group (through case manager community nurses, at subject's homes, as part of the assessment process) vs. the control group (through interviewers from the research team, members with a nursing profile, via telephone interviews) may have led to differences in handling assessment criteria, for instance, despite the fact that training for data compilation was identical for all staff taking part in this stage of the research.

To round off this section addressing the limitations of the study, it is worth pointing out that the inclusion of certain variables in relation to nurses' professional practice would have allowed for a deeper analysis of the link between interventions and outcomes, beyond the mere existence, or not, of case management as a component in care delivery. As it proved difficult to obtain data through direct observation or from nursing staff statements without incurring in bias, this idea was abandoned. This issue is to be addressed in subsequent studies by our research team using a qualitative approach.

## Conclusion

This study highlights certain relevant issues that summarise the contribution of this new home care model and its implementation in the region of Andalusia. Along with caregivers, highly dependent, immobilised subjects with high morbidity have become the target of numerous home care interventions. These have been implemented in a structured manner, using systematic assessment mechanisms that are endorsed by wide ranging scientific evidence. With such a precedent, it is very likely that future inter-sectoral or legislative initiatives will have greater social importance, leading to fairer distribution of resources.

Degrees of co-ordination and diversification are improving remarkably and it is now common to see multi-professional teams working in the home as well as harmonised resources that foster continuity of healthcare delivery. Clear proof of this is the impact on the functional capacity of patients who require home care services following hospital discharge.

Furthermore, the model provides an additional resource by rationalising demand and reducing the number of visits to healthcare centres by caregivers and patients. It also has a beneficial effect on caregiver burden and may just be the first step towards covering the many needs of these women. This issue should be addressed in future research.

The repercussion on user satisfaction of this model confirms its acceptability and provides outcome criteria for subjects receiving care.

Specific critical pathways need to be devised and implemented if we are to lower A&E visits and hospital admissions.

## Abbreviations

NOC: Nursing Outcomes Classification; PCBTs: Primary Care Basic Teams; DSM: Malaga Healthcare District; DSA: Almería Healthcare District; DSG: Granada Healthcare District; DSCS: Costa del Sol Healthcare District; GP: General Practitioner

## Competing interests

The authors declare that they have no competing interests.

## Authors' contributions

JMM-A devised the study, conducted the data analyses, and drafted the manuscript. EG-J participated in the conception, design and co-ordination of the study and assisted in the drafting of the manuscript. FJM-S participated in the design and co-ordination of the study and assisted in the drafting of the manuscript. JCM-H participated in the design and co-ordination of the study and assisted in the drafting of the manuscript. MC-M contributed with data processing, analysis and revision of the manuscript. AM-C participated in the design of the study and assisted in the revision of the manuscript. JJG-A participated in the design and co-ordination of the study as well as data processing. IT-L participated in the design and co-ordination of the study as well as data processing. All authors read and approved the final manuscript.

## Pre-publication history

The pre-publication history for this paper can be accessed here:


